# Perceptions of justice actors on juvenile fundamental rights and rehabilitation in Vietnam: findings from a multi-institutional survey

**DOI:** 10.3389/fpsyg.2025.1615065

**Published:** 2025-08-06

**Authors:** Hoang Xuan Chau, Hoang Minh Tuan, Tran Thi Tuyet Nhung, Ngoc Ha Do, Nguyen Chau Anh, Thang The Nguyen

**Affiliations:** ^1^Northern College of Law, Thai Nguyen, Vietnam; ^2^Vietnam Youth Academy, Hanoi, Vietnam; ^3^Niagara University, Lewiston, Niagara Falls, NY, United States; ^4^Faculty of Management Science, University of Social Sciences and Humanities, Vietnam National University, Hanoi, Vietnam

**Keywords:** juvenile justice, fundamental rights, rehabilitation, child-centered justice, legal professionals, institutional context, Vietnam

## Abstract

This study explores the perceptions of justice and governance professionals in Vietnam regarding the protection of fundamental rights and the implementation of community-based reintegration measures for juvenile offenders. Drawing on a cross-sectional survey of 285 respondents—including police officers, prosecutors, legal aid providers, commune officials, and other local-level actors—the research investigates three key dimensions: the perceived objectives of juvenile justice, support for a specialized juvenile justice system, and the perceived necessity of 22 child-centered, community-based interventions aimed at rehabilitation and recidivism prevention. The findings reveal strong overall support for rehabilitative and educational goals over punitive approaches. A large majority of respondents endorsed the establishment of a dedicated juvenile justice system aligned with international standards, particularly the UN Convention on the Rights of the Child. Among the proposed interventions, those involving legal safeguards, psychological support, diversion, and family engagement received the highest levels of support. While perceptions were consistent across gender, education, and professional experience, differences emerged by occupational role, suggesting competing institutional logics. These insights offer critical input for aligning Vietnam’s juvenile justice reform efforts with child rights principles and global best practices.

## Introduction

Over the past three decades, juvenile justice systems worldwide have experienced significant transformations, shifting from punitive models toward approaches rooted in child rights, rehabilitation, and community-based reintegration. This paradigmatic transition is codified in key international legal instruments, including the United Nations Convention on the Rights of the Child (United Nations, 1989), the United Nations Standard Minimum Rules for the Administration of Juvenile Justice (Beijing Rules, 1985), and the Guidelines for the Prevention of Juvenile Delinquency (Riyadh Guidelines, 1990). These instruments emphasize principles such as, the best interests of the child, proportionality, non-custodial alternatives, and the centrality of reintegration into society.

Nevertheless, the global trajectory of juvenile justice reform has not been linear. Historical and comparative research has shown that early twentieth-century juvenile courts, particularly in the United States and Europe, were initially conceived as welfare-oriented institutions designed to provide individualized and rehabilitative responses to youth offending (Feld, 1993; Garland, 2001). This philosophy, commonly described as “penal welfarism,” began to erode in the late twentieth century under the influence of rising crime anxieties, leading to what scholars have termed a “punitive turn”—a policy shift marked by increased criminalization, reduced discretion, and the incorporation of retributive principles into juvenile systems (Feld, 1998; Muncie, 2008; Pires, 2001). Recent literature, however, suggests the emergence of a new phase, in which some jurisdictions are reinvesting in restorative practices, diversion, and community-based interventions, albeit often within frameworks that retain elements of surveillance and control (Bateman, 2020; Goshe, 2023; Benekos and Merlo, 2016).

In Vietnam, the evolution of juvenile justice has mirrored aspects of these global shifts. Since the adoption of the 2015 Criminal Code and Criminal Procedure Code, the country has taken notable steps to incorporate child-specific provisions aligned with the CRC. These include enhanced legal safeguards for children in conflict with the law, as well as provisions for diversion and educational measures (Tan Duy and Dandurand, 2022). Despite these legislative advances, Vietnam has yet to establish a fully specialized juvenile justice system. Juvenile cases continue to be processed within general criminal courts, and specialized personnel or infrastructure—such as, child-sensitive procedures, trained youth judges, and inter-agency protocols—remain limited or unevenly applied across jurisdictions (Lê, 2023). Consequently, implementation of child-friendly justice principles remains fragmented and highly dependent on local capacities and institutional cultures.

Existing research on juvenile justice in Vietnam has largely focused on legal analysis or qualitative inquiries into specific case studies or institutional practices (e.g., Vinh, 2021). However, there remains a lack of empirical data capturing how justice and governance professionals—those directly engaged in the operationalization of juvenile justice policy—perceive key principles such as, rehabilitation, procedural fairness, and community-based reintegration. This gap is particularly salient at the local level, where actors such as, police officers, commune officials, legal aid providers, and youth protection staff play a pivotal role in shaping children’s experiences with the justice system.

This article addresses that gap by presenting findings from a cross-sectional survey of 285 professionals involved in Vietnam’s juvenile justice and child protection ecosystem. The study investigates three interrelated dimensions: (1) perceptions of the primary objectives of juvenile justice; (2) levels of support for the establishment of a specialized juvenile justice system; and (3) the perceived necessity of 22 child-centered, community-based interventions aimed at promoting rehabilitation and reducing recidivism. By examining these perceptions across professional roles and institutional settings, the study contributes to a more granular understanding of Vietnam’s institutional readiness to implement child rights–based justice reforms. It also situates Vietnam’s experience within broader global debates on the tensions and transitions characterizing juvenile justice systems in the Global South.

## Literature review

Over the past century, the development of juvenile justice systems has been shaped by complex and often contradictory philosophical and institutional shifts. Early reforms, particularly in the United States and parts of Europe, gave rise to what scholars have termed the penal welfare model—a framework that emphasized individualized treatment, discretionary intervention, and rehabilitative ideals (Feld, 1993; Garland, 2001). Under this model, juvenile courts were designed as protective spaces that prioritized the best interests of the child over formal adversarial procedures.

However, beginning in the 1970s and accelerating through the 1980s and 1990s, many jurisdictions witnessed what has been called the “punitive turn” in juvenile justice (Muncie, 2008; Pires, 2001). This transformation was prompted by mounting critiques of the juvenile court’s perceived leniency and lack of transparency, coupled with rising public anxieties about youth crime. As a result, systems across the United States, England, and France increasingly incorporated retributive logics, leading to the “adultification” of juvenile courts, the proliferation of mandatory sentencing, and greater emphasis on deterrence and public protection (Feld, 1998; Trépanier, 2000; Bailleau, 2005).

More recently, a growing body of scholarship has pointed to a renewed emphasis on rehabilitation and community-based alternatives, marking what some have referred to as a “restorative turn” (Benekos and Merlo, 2016; Goshe, 2023). This trend has been particularly notable in jurisdictions with strong commitments to children’s rights frameworks, such as, those influenced by the UN Convention on the Rights of the Child (UNCRC). Nonetheless, scholars have cautioned against interpreting these developments as linear or universal. As Bateman (2020) argues, rehabilitative discourses frequently coexist with punitive practices, and reforms often reflect pragmatic adjustments rather than normative consensus.

Institutionally, the orientation of juvenile justice practice varies significantly across actors. Police officers, who typically serve as first responders, often operate within frameworks of control, deterrence, and institutional efficiency. Studies have shown that police may lack adequate training in child development or procedural safeguards, leading to rights violations during arrest, interrogation, or referral decisions (Liefaard, 2015; Majeed et al., 2024). Prosecutors, for their part, have been found to act as gatekeepers of punitive logic, particularly when political or public pressures call for harsh responses to youth offending. Research from both Western and non-Western contexts has highlighted how prosecutorial discretion can contribute to the criminalization of juveniles, even when ostensibly framed in rehabilitative language (Henning, 2009; Thomas and Bilchik, 1985; Weisburd, 2015).

By contrast, judges—particularly those trained in juvenile matters—are more likely to embrace child-centered philosophies. Judicial discretion allows for the integration of contextual and psychosocial factors, and judges have historically played a central role in championing procedural safeguards and non-custodial alternatives (Kupchik, 2006; Mears et al., 2015). However, even the judiciary is not immune to systemic pressures. As Feld (1990) and Birckhead (2009) caution, institutional mandates, caseload pressures, and public sentiment can undermine rehabilitative aims and result in more punitive outcomes.

Notably, recent comparative work suggests that professional identities and institutional roles significantly shape interpretations of juvenile justice across systems. For instance, studies in India (Prasad, 2024) and Vietnam (Tan Duy and Dandurand, 2022) confirm that while judicial actors may espouse developmental rationales, law enforcement and prosecutorial agencies continue to prioritize risk management and public order, often at the expense of children’s rights.

Overall, the literature underscores the need for cross-sectoral alignment and specialized training to bridge these institutional divergences. It also highlights the importance of analyzing how contextual factors—such as, legal traditions, institutional hierarchies, and developmental state capacity—mediate the translation of international child rights norms into domestic practice (Goldson, 2011; National Research Council, 2013). Against this backdrop, the present study contributes to the literature by empirically examining how justice and governance professionals in Vietnam perceive key tenets of child-centered justice, and by identifying variations across occupational roles that may inform reform strategies.

## Method

### Study design and participants

This study adopted a cross-sectional quantitative research design aimed at exploring the perceptions of professionals involved in juvenile justice and local child governance in Vietnam. A total of 285 participants were selected using purposive sampling, ensuring diversity in geographic representation, occupational roles, and institutional affiliations. The sample included police officers (9.5%), prosecutors (1.4%), legal aid providers, commune-level officials (27.0%), neighborhood mediators, researchers, and other stakeholders involved in the handling of juvenile cases. Notably, 42.8% of respondents self-identified under the “Other” category. A follow-up review of their occupational descriptions revealed that this group comprised child protection officers, school-based legal educators, youth union representatives, and staff from local social affairs departments—actors who, though not situated within the core criminal justice system, play critical roles in the prevention, diversion, and reintegration of juvenile offenders.

Participants were recruited through existing professional networks, including provincial justice departments, legal aid centers, and youth-related organizations. The Youth Research Institute coordinated outreach efforts in collaboration with district-level People’s Committees and police units. Questionnaires were administered both in person (hard copy) and online via secure survey links between September and November 2024. All participants received formal invitations and information sheets outlining the voluntary nature of participation, confidentiality assurances, and data anonymization procedures. Ethical approval was granted by the Research Ethics Committee of the Youth Research Institute (Reference No. YRI-2024-10), and institutional support letters were issued to facilitate access.

While purposive sampling allowed the inclusion of relevant practitioners actively working with juveniles, it also limits the generalizability of findings. Moreover, the overrepresentation of commune-level personnel—who constituted more than two-thirds of the sample—reflects the operational reality of Vietnam’s juvenile justice landscape but may underrepresent perspectives from higher-level judiciary and prosecutorial actors.

### Survey instrument

The primary data collection tool was a structured questionnaire developed in accordance with international standards and national legal provisions related to juvenile justice. The instrument was informed by the UN Convention on the Rights of the Child (1989), the Beijing Rules (1985), and Vietnam’s 2015 Criminal Procedure Code. The questionnaire consisted of three main sections:

- *Section 1* collected demographic information, including gender, age, education, occupational role, institutional level, and years of professional experience;
- *Section 2* included two single-choice items: (a) the respondent’s view of the primary objective of juvenile justice, and (b) their support for establishing a specialized juvenile justice system in Vietnam;
- *Section 3* contained 22 items assessing the perceived necessity of specific child-centered, community-based reintegration measures. Each item was rated on a 3-point ordinal scale: 1 = Not necessary, 2 = Neutral, 3 = Necessary.

The instrument was reviewed by legal scholars and practitioners for content validity and piloted with a group of 15 professionals for clarity and relevance prior to full deployment.

### Reliability and composite scoring

The internal consistency of Section 3 was evaluated using Cronbach’s alpha. The resulting coefficient (*α* = 0.946) indicated excellent reliability and unidimensionality, supporting the use of composite scoring for subsequent analysis (Nunnally and Bernstein, 1994).

### Data analysis

All data were analyzed using SPSS version 25. Descriptive statistics (frequencies, percentages, means, standard deviations) were computed for all variables. Inferential analyses included independent samples *t*-tests (for gender) and one-way ANOVA (for education level, occupational role, and years of experience). Where significant group differences emerged, Tukey’s Honestly Significant Difference (HSD) *post*-*hoc* tests were conducted to identify specific pairwise contrasts. Statistical significance was set at *p* < 0.05.

## Results

### Sociodemographic characteristics of participants

The final sample comprised 285 respondents engaged in juvenile justice or child governance roles across multiple provinces in Vietnam. As shown in Table 1, the gender distribution was balanced, with 53.7% identifying as male and 46.3% as female. In terms of educational attainment, the majority held a university degree (62.8%), followed by upper secondary education (18.9%), college diploma (10.9%), and postgraduate qualifications (7.4%).

**Table 1:** Sociodemographic characteristics of participants.

Characteristic	Frequency (*n*)	Percentage (%)
Gender		
Male	153	53.7
Female	132	46.3
Education		
Upper secondary	54	18.9
College	31	10.9
University	179	62.8
Postgraduate	21	7.4
Occupation		
Police officer	27	9.5
Prosecutor	4	1.4
Commune official	77	27.0
Legal aid provider	6	2.1
Others	122	42.8
Institutional level		
Central	7	2.8
Provincial	27	10.8
District	45	18.0
Commune	171	68.4
Years of experience		
1–5 years	186	68.4
5–10 years	61	22.4
10–15 years	19	7.0
15–20 years	2	0.7
>20 years	4	1.5

Percentages may not total 100% due to rounding or missing values. Occupational categories are based on self-reported roles; “Others” include legal educators, child protection staff, and youth union officers.

Occupationally, the sample was diverse. While police officers (9.5%) and prosecutors (1.4%) represented the formal criminal justice sector, a substantial number of participants were commune officials (27.0%), legal aid providers (2.1%), lawyers, educators, and others involved in local-level justice or youth protection. Notably, 42.8% of respondents self-identified under the “Other” category, which was later determined to include child protection staff, school-based legal educators, and youth union officers.

In terms of institutional level, most respondents operated at the commune (68.4%) and district levels (18.0%), with fewer from the provincial (10.8%) and central levels (2.8%). Years of professional experience varied, with a plurality reporting between 1 and 5 years (68.4%), followed by 5–10 years (22.4%).

### Perceived purpose of juvenile justice

When asked about the primary purpose of the juvenile justice system, 44.9% of respondents identified the prevention of reoffending as its central goal, followed by education and rehabilitation (29.5%) and support and assistance for juveniles (24.6%). Only 1.1% selected punishment.

These results suggest strong endorsement of rehabilitative and preventive principles among the majority of respondents. The low selection rate for punitive responses aligns with global shifts in juvenile justice philosophy but also reflects Vietnam’s legal emphasis on reintegration as stipulated in the 2015 Criminal Procedure Code (see Table 2).

**Table 2:** Perceived primary purpose of juvenile justice.

Response category	Frequency (*n*)	Percentage (%)	Rank
Prevention of juvenile reoffending	128	44.9	1
Education and rehabilitation of juvenile offenders	84	29.5	2
Support and assistance for juvenile offenders	70	24.6	3
Punishment of juvenile offenders	3	1.1	4
Total	285	100.0	

Participants were asked to select one primary objective they believed the juvenile justice system should pursue. The majority endorsed rehabilitative and preventive approaches over punitive aims.

### Support for a specialized juvenile justice system

An overwhelming 93.0% of respondents expressed support for the establishment of a specialized juvenile justice system distinct from the adult criminal process. This high level of support indicates professional recognition of the developmental and procedural differences between children and adults in conflict with the law, and affirms the perceived inadequacy of current structures to meet the needs of juvenile offenders (see Table 3).

**Table 3:** Support for a specialized juvenile justice system.

Response option	Frequency (*n*)	Percentage (%)
Yes	265	93.0
No	20	7.0
Total	285	100.0

Respondents were asked whether they support the establishment of a separate juvenile justice system distinct from the adult criminal justice system. The overwhelming majority expressed support for such a system.

### Perceived necessity of child-centered measures

Participants rated 22 child-centered and community-based measures on a 3-point ordinal scale. Overall, endorsement levels were high: all items had mean scores above the midpoint (M > 2.5), with the highest-rated items being:

- Individual risk and protective factor assessments (M = 2.70, SD = 0.531);
- Opportunities for first-time offenders to repair harm pre-adjudication (M = 2.69, SD = 0.554);
- Use of appropriate language by justice personnel (M = 2.69, SD = 0.540);
- Provision of legal aid from the earliest stage (M = 2.68, SD = 0.521);
- Consideration of prior rehabilitation efforts during sentencing (M = 2.67, SD = 0.572).

The lowest-rated item, though still favorably viewed, was the separation of juveniles from adults in case handling (M = 2.49, SD = 0.685), indicating possible practical constraints in implementation or lower prioritization in current institutional culture.

Standard deviations were generally low, suggesting high internal agreement. The overall reliability of the 22-item scale was excellent (*α* = 0.946), affirming its psychometric robustness (see Table 4).

**Table 4:** Perceived necessity of child-centered and community-based measures.

Juvenile justice feature	Not necessary (%)	Neutral (%)	Necessary (%)	Mean	SD	Rank
Implement individual risk and protective factor assessments	3.5	23.2	73.3	2.70	0.531	1
Provide opportunities for first-time and juvenile offenders to repair harm	4.6	22.1	73.3	2.69	0.554	2
Use language appropriate for juveniles	3.9	23.2	73.0	2.69	0.540	2
Provide legal counseling and aid from earliest stage	2.5	28.1	69.5	2.68	0.521	4
Executing agencies report rehabilitation progress to court	3.2	25.3	71.6	2.68	0.529	4
Measures should help juveniles understand the harm caused	3.2	25.6	71.2	2.68	0.531	4
Provide court with prior implementation information before sentencing	5.3	22.5	72.3	2.67	0.572	6
Understand juvenile’s family, school, and community context	4.2	24.6	71.2	2.67	0.553	6
Provide family support for educating juveniles	5.6	22.8	71.6	2.66	0.581	9
Justice officials should understand and empathize with juveniles	2.5	28.8	68.8	2.66	0.523	9
Train justice officials in child psychology and juvenile justice goals	3.2	29.1	67.7	2.65	0.541	11
Establish early community interventions for at-risk youth	4.2	26.3	69.5	2.65	0.559	11
Assign social workers to accompany juveniles throughout the process	4.2	28.1	67.7	2.64	0.563	13
Procedures should involve juveniles in their own case resolution	5.3	26.7	68.1	2.63	0.583	14
Conduct interviews in private settings	8.1	22.1	69.8	2.62	0.632	15
Diversify community-based sanctions to avoid isolation	4.6	29.1	66.3	2.62	0.573	15
Juveniles should participate in restorative justice processes	3.5	31.6	64.9	2.61	0.555	17
Child-friendly courtroom and interview environments	7.0	26.7	66.3	2.59	0.619	18
Prioritize community-based solutions, detention as last resort	10.5	26.3	63.2	2.53	0.679	19
Maximize use of community resources and diversion	7.4	32.3	60.4	2.53	0.631	19
Victims should participate in resolution processes	9.8	29.5	60.7	2.51	0.669	21
Not handling juveniles together with adults	10.9	29.5	59.6	2.49	0.685	22

Respondents rated the necessity of 22 juvenile justice measures on a 3-point ordinal scale (1 = Not necessary; 2 = Neutral; 3 = Necessary). Items are ranked by descending mean score. SD, Standard Deviation.

## Group differences

### Gender

An independent samples *t*-test revealed no statistically significant gender differences in mean support for child-centered interventions (Male: M = 2.615, SD = 0.410; Female: M = 2.645, SD = 0.384; *t*(283) = −0.627, *p* = 0.531). This suggests that gender did not significantly influence respondent perceptions.

### Education and experience

One-way ANOVA indicated no significant differences in composite support scores by education level (*F*(3, 281) = 1.656, *p* = 0.177) or years of experience (*F*(4, 276) = 1.388, *p* = 0.238). This consistency suggests a broadly shared normative orientation across professional tenure and academic background.

### Occupational role

In contrast, significant variation was observed across occupational groups (*F*(10, 274) = 2.768, *p* = 0.003). *Post-hoc* Tukey HSD tests revealed that commune officials and social sector professionals were more likely to endorse child-centered measures than police or legal professionals. This divergence underscores the influence of institutional mandates on professional attitudes—confirming findings from comparative research (e.g., Kupchik, 2006; Prasad, 2024).

### Summary of key findings

1. There is widespread support among local justice and child governance actors for rehabilitative objectives in juvenile justice, with punishment receiving negligible endorsement.
2. The vast majority of respondents support the development of a specialized juvenile justice system.
3. All 22 proposed child-centered interventions were viewed as necessary by most participants, with especially high support for individualized assessment, legal aid, and restorative practices.
4. While perceptions were consistent across gender, education, and experience, occupational role significantly influenced views, indicating the presence of competing institutional logics within the broader system.

## Discussion

This study provides an important empirical contribution to understanding how professionals engaged in juvenile justice and local child governance in Vietnam interpret the principles of child-centered justice. The results point to widespread normative support for rehabilitation, prevention of reoffending, and the development of a specialized juvenile justice system. However, the analysis also reveals significant variations across professional groups and institutional contexts, underscoring the need for a more differentiated and critically reflective approach to reform.

### Divergent institutional logics: beyond the illusion of consensus

While the majority of respondents favored prevention and rehabilitation over punishment, the data indicate that this orientation is not uniformly distributed across occupational roles. Police officers and legal professionals, though part of the justice system’s formal apparatus, expressed comparatively lower levels of endorsement for several key child-centered interventions—such as, diversion and family-based measures—than social sector and local governance actors. These patterns echo existing international research, which has documented how institutional mandates and occupational cultures shape perceptions and practices in youth justice systems (Kupchik, 2006; Henning, 2009; Prasad, 2024).

As scholars such as, Feld (1993, 1998) and Muncie (2008) have argued, police and prosecutors often operate within logics of deterrence, control, and accountability, which may conflict with restorative or developmental approaches. Even where rehabilitative ideals are present in legal frameworks, frontline discretionary practices are frequently governed by institutional performance metrics and public pressure. In contrast, social protection workers, legal educators, and commune officials are more likely to prioritize reintegration, family engagement, and individualized support, given their proximity to community-based care and non-custodial mechanisms.

### Professional support vs. structural readiness

The near-universal support (93%) for a dedicated juvenile justice system suggests a shared recognition of the inadequacy of current arrangements. Yet such support, while meaningful, must be interpreted with caution. As Bateman (2020) and Goshe (2023) observe, rhetorical alignment with international standards often coexists with persistent structural barriers—including insufficient personnel, lack of inter-agency coordination, and entrenched punitive tendencies. In Vietnam, despite the presence of legal provisions in the 2015 Criminal Code and Procedure Code, the absence of a fully operational specialized system means that juvenile cases continue to be processed through adult criminal courts, often without the benefit of dedicated facilities or trained staff (Tan Duy and Dandurand, 2022; Lê, 2023).

Moreover, the lower rating of measures such as, the separation of juveniles from adults during case processing (Mean = 2.49) raises concerns about institutional feasibility, even when professionals express normative support. This discrepancy between attitude and anticipated practice highlights the tension between ideals and operational realities—a dynamic well-documented in studies of juvenile justice reform across Global South contexts (Goldson, 2011; National Research Council, 2013).

### Occupational differences and implications for reform design

One of the study’s most significant findings is the statistically significant variation in support for child-centered measures by occupational role. This reinforces the importance of tailoring reform strategies to specific professional contexts, rather than assuming a uniform set of priorities or values across the justice system. For instance, capacity-building programs for police and prosecutors may need to foreground child rights, developmental psychology, and procedural safeguards, while initiatives for commune-level officials may emphasize coordination mechanisms and early prevention strategies. Such differentiation is critical to fostering sustainable, cross-sectoral buy-in for reform.

It is also worth noting that gender, education, and years of experience did not significantly affect perceptions, suggesting that occupational identity—more than demographic profile—shapes normative orientations in juvenile justice. This finding supports a growing body of literature emphasizing the salience of institutional cultures in shaping professional behavior (Mears et al., 2015; Sprong et al., 2017).

### Methodological reflections and contextual limitations

While the study offers a rare quantitative snapshot of professional attitudes, several limitations must be acknowledged. First, the use of purposive sampling limits the generalizability of the findings. The sample was disproportionately composed of commune-level personnel (over 68%), and the representation of prosecutors (1.4%) and judges (0%) was minimal or absent. This raises questions about how far the findings can be extrapolated to the broader justice system.

Second, the category “Other”—which encompassed over 40% of respondents—reflects the diffuse institutional architecture of juvenile justice in Vietnam, where case handling often involves actors outside the core criminal justice apparatus. While this may be viewed as a limitation in analytical precision, it also reflects the reality of decentralized responsibility and the intersectoral nature of youth justice in practice.

Third, the cross-sectional nature of the survey captures professional attitudes at a single point in time. Future research should consider longitudinal and mixed-methods approaches to better understand how these attitudes evolve, how they translate into actual behavior, and how they interact with institutional reform trajectories.

### Toward context-sensitive reform

In sum, the findings of this study affirm the relevance of international juvenile justice principles in the Vietnamese context but also expose the complexity of translating normative consensus into effective institutional change. Reform efforts must move beyond legal alignment and attend to the everyday practices, constraints, and values of those charged with implementation.

For Vietnam, a context-sensitive reform strategy would include the establishment of specialized juvenile units at the district level, the expansion of non-custodial and community-based sanctions, and the institutionalization of inter-agency cooperation. Equally important is sustained investment in professional training tailored to specific institutional roles.

By highlighting both convergence and divergence in professional perceptions, this study offers a critical evidence base for shaping juvenile justice reform in Vietnam and contributes to broader global debates on the challenges of building child-friendly justice systems in transitional legal settings.

## Conclusion

This study provides empirical evidence of broad professional support in Vietnam for shifting the juvenile justice system toward a child-centered, rehabilitative model aligned with international human rights standards. Most participants endorsed the principles of rehabilitation, reintegration, and diversion over punishment, and expressed strong support for the establishment of a specialized juvenile justice system.

However, this consensus is not uniform across institutional roles. Occupational differences—particularly between law enforcement and social governance actors—suggest the existence of competing institutional logics. These differences must be acknowledged in policy design to avoid assuming a one-size-fits-all reform trajectory. Moreover, the prevalence of commune-level actors in case handling highlights the importance of investing in local capacities and cross-sector collaboration.

Based on the findings, the following policy recommendations are proposed:

1. Establish a dedicated juvenile justice system, with clear legal mandates, child-friendly infrastructure, and specialized personnel, particularly at the district level where most juvenile cases originate.
2. Integrate child rights and developmental psychology into training curricula for police officers, prosecutors, and other frontline justice actors to promote a shared rehabilitative ethos.
3. Strengthen the role of community-based services, including social work, legal aid, and restorative practices, to reduce reliance on custodial measures and support reintegration.
4. Institutionalize inter-agency coordination mechanisms, especially between justice, education, and social affairs sectors, to ensure holistic responses to youth offending.
5. Develop monitoring frameworks that assess not only legal compliance but also the consistency of institutional practices with child rights principles.

In sum, while legal reforms in Vietnam have laid important groundwork, their meaningful implementation requires targeted investment, institutional restructuring, and sustained professional development. Bridging the gap between normative support and operational practice will be essential to realizing a truly child-friendly justice system.

## Data Availability

The original contributions presented in the study are included in the article/supplementary material, further inquiries can be directed to the corresponding author.
